# Thymoquinone: Hydroxypropyl-β-cyclodextrin Loaded Bacterial Cellulose for the Management of Wounds

**DOI:** 10.3390/pharmaceutics14122816

**Published:** 2022-12-15

**Authors:** Sam Swingler, Abhishek Gupta, Hazel Gibson, Marek Kowalczuk, Grazyna Adamus, Wayne Heaselgrave, Iza Radecka

**Affiliations:** 1 Department of Biology, Chemistry and Forensic Science, School of Sciences, Faculty of Science and Engineering, University of Wolverhampton, Wolverhampton WV1 1LY, UK; 2 School of Allied Health and Midwifery, Faculty of Education, Health and Wellbeing, University of Wolverhampton, Walsall WS1 3BD, UK; 3 Centre of Polymer and Carbon Materials, Polish Academy of Sciences, M. Curie-Sklodowskiej 34, 41-819 Zabrze, Poland; 4 Department of Biomedical Science, University of Wolverhampton, Wolverhampton WV1 1LY, UK

**Keywords:** biomaterials, experimental dermatology, controlled drug release, biopolymer, anti-fungal, wound dressing, bacterial cellulose

## Abstract

The need for more advantageous and pharmaceutically active wound dressings is a pressing matter in the area of wound management. In this study, we explore the possibility of incorporating thymoquinone within bacterial cellulose, utilising cyclodextrins as a novel method of solubilising hydrophobic compounds. The thymoquinone was not soluble in water, so was incorporated within hydroxypropyl-β-cyclodextrin before use. Thymoquinone: hydroxypropyl-β-cyclodextrin inclusion complex produced was found to be soluble in water up to 7% (*w*/*v*) and was stable with no crystal formation for at least 7 days with the ability to be loaded within the bacterial cellulose matrix. The inclusion complex was found to be thermally stable up to 280 °C which is far greater than the production temperature of 80 °C and was stable in phosphate-buffered saline and extraction solvents in permeation and dose experiments. The adhesion properties of the Thymoquinone: hydroxypropyl-β-cyclodextrin loaded bacterial cellulose dressings were tested and found to be 2.09 N. Permeation studies on skin mimicking membrane Strat-M showed a total permeated amount (0–24 h) of 538.8 µg cm^−2^ and average flux after a 2 h lag of 22.4 µg h^−1^ cm^−2^. To the best of our knowledge, the methods outlined in this study are the first instance of loading bacterial cellulose with thymoquinone inclusion complex with the aim of producing a pharmaceutically active wound dressing.

## 1. Introduction

The rate at which wounds heal is influenced by many factors, including the size, type, depth and whether there is an infection already present. Wound healing is a highly complex physiological process. Many steps can overlap, and the order in which they occur is equally intricate [1,2].

The local environment and wound site can be separated using established dressings such as gauze and tulle. However, being passive in nature, they are not able to facilitate the healing process of wounds directly [3,4,5]. On the other hand, moist wound dressings, such as bacterial cellulose (BC) dressings, being interactive in nature facilitate healing by keeping the wound optimally moist. Among the advantages of these dressings is that they are easily removed from the wound site, allowing the dressing to be changed without causing further trauma to the patient [1,6,7,8,9].

Furthermore, these hydrogel dressings react to variations in moisture levels, which facilitates the re-epithelialization of the wound site [1]. Advanced hydrocolloid and hydrogel dressings do more than absorb and retain excess wound exudate; they also provide a cooling, soothing effect, thereby reducing blushing and pain perception [10]. However, whether a wound dressing is wet or dry, if infection is present around or in the wound, microbes can thrive in the moist environment created by the dressing or wound exudate, thus leading to an aggravated healing process [11,12].

In recent years, advancements in biotechnology has led to the production of biosynthetic materials with potential biomedical applications and bacterial cellulose (BC), produced by obligate aerobes such as *Gluconacetobacter xylinus* is a good example. These biosynthetic materials are attracting wide interest in their application as advanced wound dressing [13,14]. After successful trials, BC has gained much attention in the medical sector, especially for the treatment of burns. Researchers have found that burns treated with biosynthetic microbial cellulose (another name for BC) dressing showed rapid re-vascularisation and re-epithelialisation and had fewer scars than traditional treatments [15]. It is of specific interest because, unlike plant cellulose, BC is ultra-pure and free of various contaminants such as lignin, pectin, and hemicellulose [16]. Furthermore, because of the irregular crystal structure, the microfibers form a web-like structure with a much higher tensile strength than plant cellulose [17].

The properties of bacterial cellulose, including biocompatibility, non-pyrogenicity, and hydrophilicity, make it an ideal material for wound treatments [18]. The hydrophilic properties of BC and its relatively high swelling ratio make it effective at maintaining a moist environment at the wound site while maintaining a constant temperature, with an average water content of 99% [19]. As a result of BC’s hydrophilicity, BC based dressing can reversibly swell and de-swell, which is potentially helpful for treating exudates from heavily emanating wounds [20].

Thymoquinone (THQ) is the main bioactive compound in the volatile oil of *Nigella sativa*, which belongs to the Ranunculaceae family. Many Middle Eastern countries use *N. sativa* seeds for dietary purposes, commonly known as black cumin seeds. In 1963, El–Dakhakhny first extracted THQ (30–48%) from *N. sativa* seeds and reported that this component was responsible for its biological effects [21,22,23]. Generally recognised as safe (GRAS) by the Food and Drug Administration (FDA), *N. sativa* oil exerted major pharmacological activities, including anti-convulsant, anti-microbial, anti-cancer, anti-histaminic, anti-diabetic, anti-inflammatory, and antioxidative effects [23,24,25,26,27]. The presence of the compound elicits potent antioxidant activity as a result of its ability to scavenge free radicals and increase the transcription of genes involved in the production of various antioxidants, such as super-oxide dismutase (SOD), catalase, and glutathione peroxidase (GSH) [23,24,25].

In our previous studies the antimicrobial activity, as well as toxicity, both thymoquinone and hydroxypropyl-β-cyclodextrins (HPβCD), were shown to be well within accepted limits for use as a topical antimicrobial [1,2]. However, THQ ‘s highly hydrophobic nature limits a variety of biomedical applications [24,25,26]. To this end, this feasibility study shows how THQ was formed into a soluble THQ:HPβCD-inclusion complex (THQ:HPβCD-IC) to overcome problems of solubility and loading. It is presented in this feasibility report that cyclodextrin was used to solubilise the API (THQ), and thus enable loading into BC to produce hydrogels (water-based) for wound management.

## 2. Materials and Methods

### 2.1. Media and Reagents

Phosphate buffered saline (PBS, pH7.4) was purchased from Sigma-Aldrich (Gillingham, UK). Dimethyl sulfoxide (DMSO) HPLC grade, sodium hydroxide, disodium phosphate, citric acid and acetone were purchased from Sigma-Aldrich (Irvine, UK). Dextrose, bacteriological peptone, yeast extract, Hestrin and Schramm agar (HSA), Hestrin and Schramm media (HS) were purchased from Lab M (Bury, UK). HPLC gradient grade water, methanol, poly(ethylene glycol) (PEG400), and trifluoroacetic acid (TFA) was purchased from Fisher Scientific (Cramlington, UK). All media and reagents were prepared according to the manufacturer’s instructions.

### 2.2. Microorganisms

LGC Standards Ltd. (Teddington, UK) provided *Gluconacetobacter xylinus* ATCC^®^ 23,770 for analysis. Prior to use, the organism was obtained in lyophilised form and kept at a temperature of 20 °C. In order to revive *G. xylinus*, sterile HSA was used at 37 °C for five days [1]. To prevent the bacterial cells from conglomerating into a single pellicle, overnight cultures of stock plates were grown under agitation in HS broth medium at 37 °C for 24 h.

### 2.3. Active Pharmaceutical Ingredient (API) Extraction

The dose of API (THQ) in prepared formulations was determined by extraction experiments. 12 replicate samples (0.5 cm^2^) were submerged into 10 mL of 50:50 acetonitrile: water (*v/v*) for 24 h and then assayed using the validated HPLC method (see Section 2.9). The mass of API in each analyte was calculated employing the below Equation (1) [1].

Dose = (A − I)/S × V(1)

(Equation (1)) where A—integrated area of peak (HPLC), I and S—intercept and slope of the calibration curve, V—volume of the acceptor medium.

### 2.4. Production and Purification of Bacterial Cellulose

BC production was conducted according to the protocol described in our previous paper [2,28]. A starter culture of *G. xylinus* was inoculated into HS media and incubated at 37 °C for 14 days in this study. In the following days, BC pellicles that had formed on the surface of the HS medium were harvested aseptically. As a preliminary step, unpurified BC was heated to 100 °C in distilled water, followed by an addition of 2% sodium hydroxide (*w*/*v*), and reheated to 100 °C in fresh distilled water for up to an hour, or until full transparency was achieved. The BC was then further washed in distilled water to ensure complete removal of sodium hydroxide and neutralise pH. After freezing each pellicle at −20 °C, it was lyophilized [1].

### 2.5. Preparation of THQ:HPβCD-Inclusion Complex

THQ:HPβCD-IC was synthesised by the solvent evaporation (SE) method following the protocol previously reported [20,28]. Briefly, THQ solution (0.8 g in 37 mL acetone) was added dropwise to the aqueous HPβCD solution (3.0 g in 12.5 mL de-ionized water) under constant agitation at room temperature. The mixture was continually stirred for a further 48 h in a container covered with perforated aluminium foil to allow for slow and complete evaporation of acetone. The sample was centrifuged at 3000 RPM for 20 min at room temperature, and the aqueous supernatant containing the solubilised THQ:HPβCD-IC was frozen after filtration using a 0.2 µm filter and lyophilised. All remaining undissolved THQ was discarded [20].

### 2.6. Preparation of Formulations 

All formulations were produced using mass fractions of ingredients. Each component was accurately weighed on an analytical balance. Masses of liquid components were converted into volumes and added using calibrated pipettes. An example of THQ:HPβCD-BC formulation is given in Table 1.

The BC pellicles remained dry, up until the loading process whereby they absorbed the aqueous THQ:HPβCD solution and were then classed as wet. Loading was performed under contact agitation at 120 rpm at room temperature.

### 2.7. Thermal Degradation

The thermal stability of API, HPβCD and THQ:HPβCD was determined. Thermogravimetric analysis (TGA) was utilised to identify the temperature of decomposition using a Mettler Toledo Thermogravimetric Analyzer, TGA/DSC 1 STAR^®^ System, Leicester, UK [20].

Forced degradation studies were also performed to determine the stability of THQ:HPβCD during curing conditions. Samples of THQ:HPβCD were stored in an oven in the presence of oxygen at 80 °C for periods of 1, 2, 3, 4, 6, 8 and 24 h. Samples were removed from the oven at the stated time points and dissolved in 10 mL of 50:50 acetonitrile:water (*v:v*) and analysed by High Performance Liquid Chromatography (HPLC).

### 2.8. Fourier Transform Infrared Spectroscopy (FTIR)

Purified BC and BC loaded with THQ:HPβCD were cut into 5 mm disks and lyophilised. The dried BC disks were then analysed using a Bruker ATR FTRI spectroscope after running background scans (Bruker, Billerica, MA. USA). Each loaded BC sample was analysed in triplicate twice and was scanned 16 times at 400–4000 cm^−1^ at room temperature [1].

### 2.9. Permeation Studies

In order to apply the samples to the synthetic human skin membranes (Strat-M) at ambient temperature, samples were cut to size using a die cut punch of 5 mm. These were then placed into jacketed diffusion cells (A = 0.5 cm^2^). The acceptor solution (PBS:PEG400 60:40 *v/v*) was equilibrated at 32 °C for 15 min while maintaining the jacket temperature at 36 °C prior to the start of the experiment. The acceptor medium was stirred at a constant speed of 200 revolutions per minute throughout the experiment. A validated HPLC method was used to analyse samples at regular intervals using a validated method (see Supplementary Materials S1). For each formulation, three diffusion cells were employed simultaneously. A median value of six measurements (*n* = 6) was used to calculate the cumulative and flux values. After the permeation study period membrane and dressing samples were immersed in 10 mL of acetonitrile:water (50/50 *v/v*) for 24 h to extract any residual API. Obtained values were further processed and presented as mean and median values [1].

### 2.10. Adhesion Testing 

The adhesion of the dressings was determined using a Mecmesin MultiTest-I Single-Column Force Tester, PPT Group UK Ltd., Sussex, UK. The instrument was set up in accordance with the manufacturer’s guidelines to perform 90 degree peel tests. Stainless steel is employed as the substrate. The method uses a speed of 100 mm per minute to ensure that a 90 degree angle is maintained during the test. Samples were cut to 9 cm × 2.5 cm. An average of three measurements (*n* = 3) was accepted as a statistically robust value of adhesion. All methods were developed according to Standard Test Method for 90 Degree Peel Resistance of Adhesives D6862-11(2021) [29].

### 2.11. Crystal Growth

To evaluate the stability of THQ:HPβCD dissolved, a crystal seeding experiment was performed. A small sample of solid THQ:HPβCD was placed on the surface of a sample of each wet dressing and stored at room temperature for 1 week. The area of crystal seeding was observed daily using a microscope. The solid THQ:HPβCD applied to the matrix of the BC induces the formation of further crystals and the greater the saturation of THQ:HPβCD in the formulation the greater the likeness of further crystals forming [29].

### 2.12. Statistical Analysis

In this study, all experiments were performed in triplicate twice, and all data presented are means accompanied by standard deviations (SD) unless stated otherwise. In each experiment, the data were analyzed using one-way ANOVA post hoc Tukey (GraphPad Prism V. 9.0.1(151), GraphPad Software, San Diego, CA, USA) with a significance level of (*p* ≤ 0.05).

## 3. Results and Discussion

Following successful biosynthesis of BC using the method described in Section 2.4, all BC was thoroughly washed and sterlised by autoclaving. Before purification, due to biomass and culture media, BC pellicles were brownish in appearance. Purified, unloaded (neat) BC was transparent confirming the removal of remnants of biomass and culture media. This is in accordance with our findings previously reported [2,20,28]. Due to the pigmentation of THQ:HPβCD, the loaded BC turned translucent, but was bright yellow in colour, suggesting successful loading of the inclusion complex. Similar findings are reported by our group with curcumin-loaded BC where the change in colour of BC after loading with curcumin [20].

THQ has been shown in several studies to possess a myriad of health benefits, but most relevant to this study is the antimicrobial properties. In our previous paper, we explored the anti-fungal properties of THQ, however, other studies have demonstrated multifunctional properties like anti-bacterial, anti-oxidant and anti-inflammatory activities which are all ideal in the treatment of wounds [30,31,32]. Nonetheless, due to the highly insoluble nature of THQ, it is extremely difficult to utilise in a clinical setting, without modifying either the chemical structure potentially rendering the molecule inactive, or by encapsulating the compound in a highly soluble inclusion complex, as used in this study, HPβCD. Similar attempts were reported by Al-Qubaisi et al., (2019) where the authors employed ultrasonication and mixing technique for encapsulation of THQ in HPβCD with the aim to improve its biopharmaceutical attributes [33]. In the present study, solvent evaporation method was employed to achieve IC formation. Attributing to the therapeutic benefits of THQ and considering its hydrophobic nature, Algahtani et al., (2021) reported the production of THQ loaded nanoemulgel with enhanced topical efficacy for wound healing applications [26].

### 3.1. Solubility of API

The API was in the form of thymoquinone hydrochloride which is insoluble in water. The thymoquinone hydrochloride was encapsulated in HPβCD and converted into THQ:HPβCD-IC by solvent evaporation method described in Section 2.5. This was a particularly crucial step in the process as the THQ needed to be solubilised in order to successfully load the IC into the BC [33,34,35,36].

THQ:HPβCD was found to be molecularly soluble in water up to 7% (*w/v*) without the addition of any excipients. The time taken for THQ:HPβCD to fully dissolve was over 2 h even when the loading was reduced to 5% (*w/v*). These results are comparable to published data into the complexation of THQ with HPβCD, however the present studies utilized various excipients which are novel to this application [37,38,39,40].

In order to reduce the time taken for complete dissolution of thymoquinone a co-solvent, DMSO, was utilised, and added at 10 wt% which reduced loading time. With the introduction of DMSO, the homogenisation time reduced to 45–60 min for complete dissolution of THQ:HPβCD, a reduction of 50–62.5% compared to diethylene glycol monoethylether (DEGEE) and with no co-solvent, respectively (Table 2).

Formulation THQ.010 was selected for further optimisation work due to shorter mixing times. Formulations containing 7.0 wt% of THQ:HPβCD were also to be assayed once a final combination of excipients is selected. The drug delivery performance of all formulations was compared to the flux values of THQ.010. Although thymoquinone with DMSO was loaded in BC as a standalone API in our previous paper [2] with a high level of antimicrobial activity, the combination of THQ:HPβCD and BC has, to the best of our knowledge never, been performed before. Nevertheless, as thymoquinone and cyclodextrins have been shown to possess minimal to no toxicity at therapeutic levels, it can be assumed that the combination of both would yield similar results.

Before loading in BC, the successful complexation of THQ and HPβCD was confirmed by different analytical methods. Additionally, the products and reactants, both prior and after synthesis, were measured to estimate percent yield (% yield). After filtration, HPβCD being water soluble at room temperature, should be in the solution form, however, there was around 0.39 g of thymoquinone that precipitated out of the acetone back into a insoluble crystal. This indicates that there is a potential for around 89.74% yield of THQ:HPβCD which was successfully obtained. These findings suggest 1:1 molar ratio of THQ and HPβCD which is in accordance with literature [33]. Further characterisation of the complex will be conducted, in future studies, to determine the exact amount of THQ that has been loaded.

### 3.2. Thermal Stability of API

**Figure 1:**
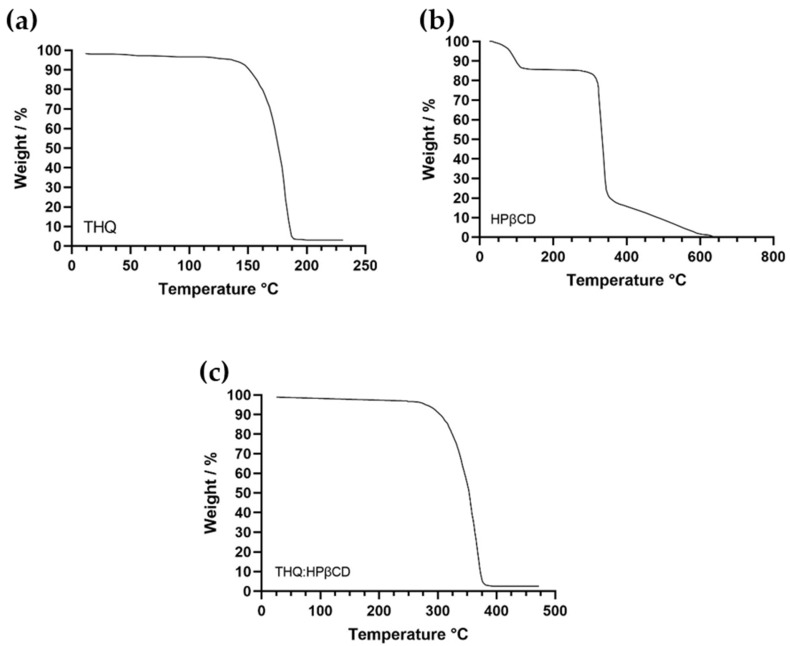
(**a**–**c**). Thermogravimetric analysis (TGA) of controls (**a**) THQ, (**b**) HPβCD, and sample THQ:HPβCD-IC (**c**) showing the thermal stability.

The thermal stability of THQ (API) was determined using thermogravimetric analysis (TGA) to understand thermal decomposition and to ensure that the API was thermally stable. The results revealed that THQ:HPβCD was stable up to 280 °C (Figure 1).

TGA analysis of HPβCD and THQ:HPβCD was also performed. TGA results from control studies revealed that THQ is thermally stable up to 150 °C (Figure 1a), while HPβCD was stable up to 300 °C. TGA thermograms for all samples (Figure 1a–c) demonstrated 2-step thermal degradation process. The first minimal weight loss (%) in differential thermograms was attributed to the trapped moisture. The second weight loss (%) is major which was reveled in the form a dip representing thermal degradation of samples. These results are in accordance with literature and our previously reported finding [20,33]. The results revealed that the complexation of the two components (THQ and HPβCD) reduced the thermal stability of HPβCD by 6.9%, however, increased the overall stability of THQ by 60.47%. This coincides with available data from previous studies investigating TGA of THQ and HPβCD, with thermal degradation of the compounds showing a reduction at 213 °C and 300 °C, respectively [41,42,43,44,45].

**Figure 2:**
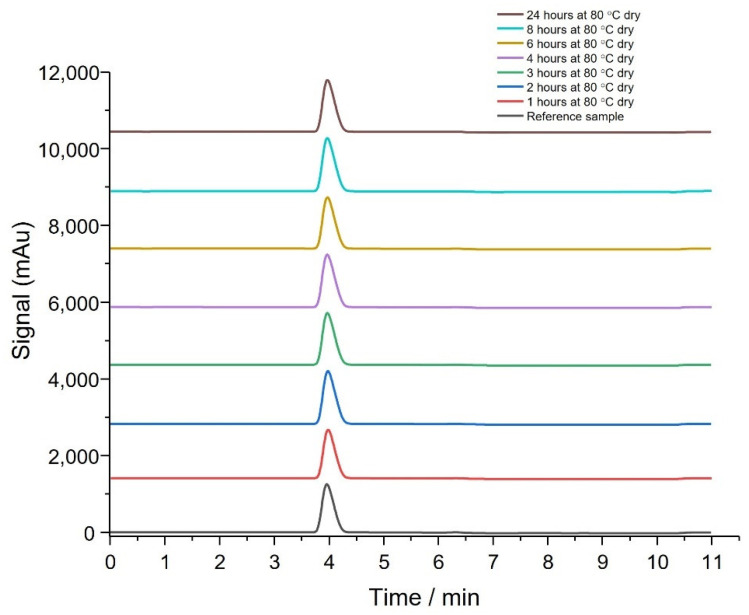
HPLC analysis showing the thymoquinone: hydroxypropyl-β-cyclodextrin was stable after forced heat degradation (dry) (*n* = 6).

Forced degradation studies were performed according to the above method. Obtained chromatograms were compared to a fresh reference sample prepared at room temperature (Figure 2). The thymoquinone eluted at the 4 min retention time irrespective of degradation time and no impurity peaks were observed confirming that THQ is thermally stable as no degradation was evident. This confirms thermal stability of THQ at 80 °C over 24 h.

**Figure 3:**
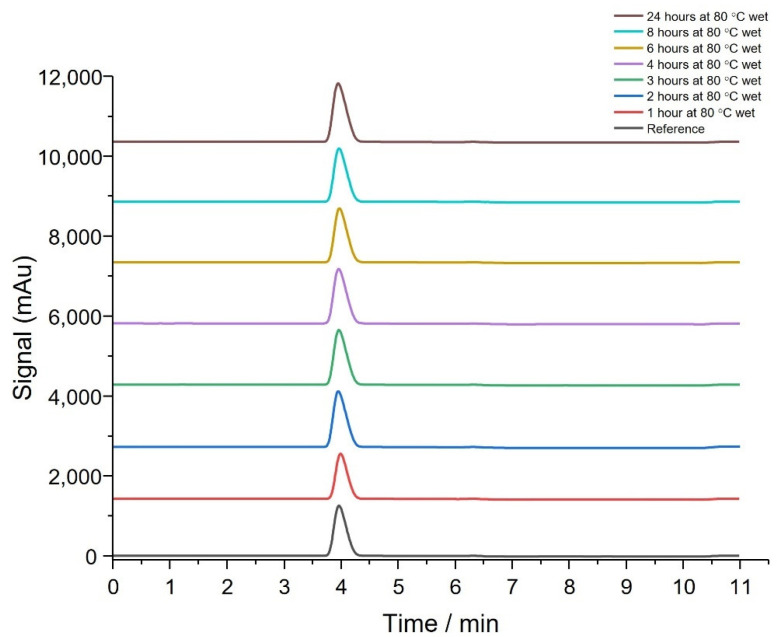
HPLC analysis showing the thymoquinone: hydroxypropyl-β-cyclodextrin was stable after forced heat degradation (wet) (*n* = 6).

The stability of THQ:HPβCD in the presence of water was also investigated. Samples of THQ:HPβCD (10 mg) were dispersed in 5 mL of water and kept in the same conditions as the dry experiment above. Once samples were taken at each time point, 5 mL of acetonitrile was added to give a 10 mg sample dissolved in 10 mL of 50:50 acetonitrile:water (*v*:*v*). Analysis of the wet degradation samples showed THQ:HPβCD eluted at 4 min and no impurity peaks observed (Figure 3). The results obtained are similar to data found from previous studies- however, in this study, no pharmaceutical grade impurities could be obtained to run alongside the test samples. This could result in some degradation species being unidentified- however this should not impact the overall thermal stability potential of the THQ:HPβCD [33].

Thermal degradation studies and TGA analysis have shown that THQ:HPβCD is stable at 80 °C for 24 h regardless of being wet or dry (Figure 2 and Figure 3). The presence of water had little effect on degradation of THQ:HPβCD which showed the API would be stable during the moisture curing process. This is important to evaluate that the overall stability of the THQ:HPβCD loaded BC dressing is stable for a prolonged period of time at room temperature, thus would be stable during packaging and storage [43,46] of the actual wound dressing for the recommended 12 month period as required by Medicines & Healthcare Products Regulatory Agency (MHRA). The current study needs to be extended to satisfy these standards prior to their potential proprietary application.

### 3.3. FTIR Confirmation

A detailed investigation on the vibrational spectra of THQ, HPβCD and THQ:HPβCD-IC was undertaken to establish the inclusion of THQ in HPβCD cavity. In our previous study, FTIR was used to establish the inclusion complex formation [20].

**Figure 4:**
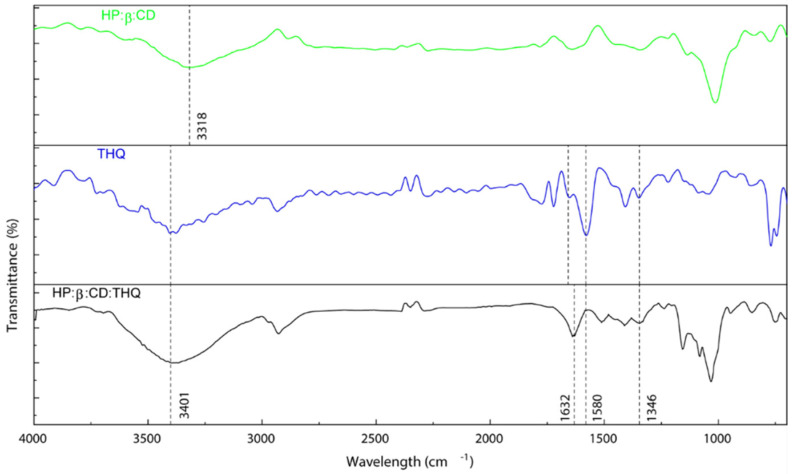
FTIR of controls HPβCD, THQ and sample THQ:HPβCD highlighting key peaks.

Figure 4 show FTIR spectra of HPβCD, THQ and THQ:HPβCD, respectively. In the case of HPβCD, the characteristic broad band of OH group stretching was shown at 3318 cm^−1^ and C-O-C glucose unit stretching occurred at 1020 cm^−1^ was detected. The FTIR spectrum of THQ shows the intense sharp band at 1647 cm^−1^, due to the carbonyl stretching, the band at 2970 cm^−1^ resulting from CH_2_ stretching vibration as well as additional bands of thymoquinone in the infrared region 1450−1020 cm^−1^, due to the C=C bending vibration (aliphatic) and C-O-C stretching vibration were detected. The infrared spectrum of THQ:HPβCD reflects a substantial variation in characteristic thymoquinone bands in particular the infrared bands in the 1350–1800 cm^−1^ region. The most interesting signals for probing the interaction between thymoquinone and HPβCD seem those, related to the polar functional groups of thymoquinone and HPβCD. The shift and gradual disappearance of the thymoquinone bands, in particular at 1647 cm^−1^ due to the carbonyl stretching and shift of the characteristic broad band of the OH groups of HPβCD from 3318 cm^−1^ to 3401 cm^−1^ can indicate that the thymoquinone was entrapped in the HPβCD cavities and thus confirm of the inclusion complex formation.

These results coincide with previously published studies that corroborate our findings. Similar spectra were observed by Al-Qubaisi et al. in their TGA analysis of THQ with Cardoso et al., 2012 also observing signature peaks between 3320 cm^−1^ to 3400 cm^−1^ provide further evidence that the THQ in this study was successfully encapsulated within the HPβCD [33,38].

### 3.4. API Permeation and Extraction

**Figure 5:**
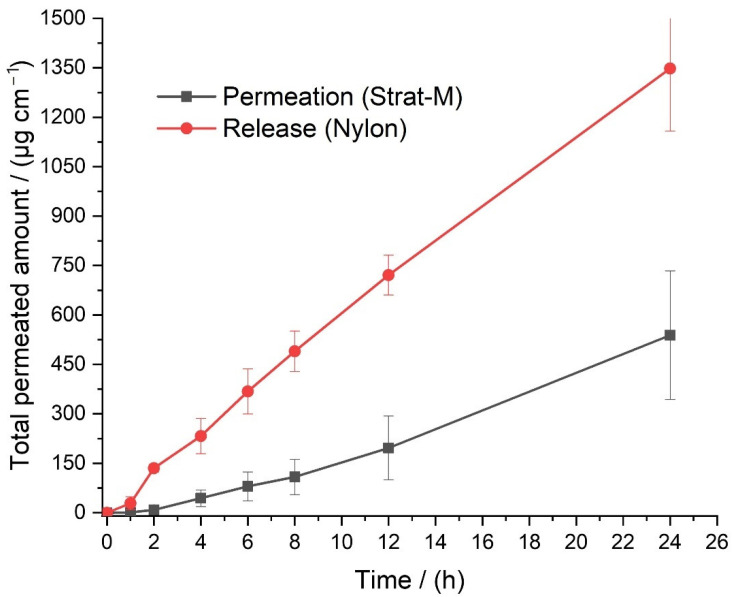
Permeation of THQ:HPβCD through Strat-M compared with release through a Nylon membrane (*n* = 4), error bar= standard error (SE).

To determine if a THQ:HPβCD:BC dressing is effective as a transdermal delivery system the permeation through a skin mimicking membrane Strat-M was tested (Table 3, Figure 5). The total released amount was plotted against the permeated amount through Strat-M to determine the rate limiting effect of the skin mimicking membrane.

The rate of permeation is lower for the skin mimicking membrane compared to Nylon which has no limiting properties (Figure 5). There is also a 2 h lag time between the application of a dressing and permeation of thymoquinone into the acceptor solution. For a basic formulation this is evidence that thymoquinone can be delivered transdermally and a dressing would be a viable method of thymoquinone delivery. Further development and optimisation will be addressed in a future study where formulations can be further modified in order to achieve higher flux by changing stoichiometric values of the formulation mixture. The importance of determining the permeability of the API is crucial in establishing whether the complex is suitable to be used in a transdermal application in wound dressings. As presented above, the results are conducive with previous studies and acceptable limits showing that THQ:HPβCD fluxes out of the BC dressing and through a skin mimicking membrane at an acceptable therapeutic rate [26,47].

The dose uniformity of the API per area depends on two factors: the homogenisation of the formulation in the mixing vessel and the control over the thickness of the API-in-BC layer. Following dose uniformity assays, the dose uniformity was as expected as seen in Table 4. The API was extracted to determine whether there was an effective dose of API within the dressing. If too little API was present, the dressing would have been rendered ineffective, and if there was too much, adverse effects may present in the user. The results obtained ($\bar{x}$ 3.31 mg cm^−2^) fall within the expected dose of THQ [46,47,48]. The calculation of the mean dose in mg cm^−2^ has been adjusted to account for the 0.5 cm disk used in the assay.

**Figure 6:**
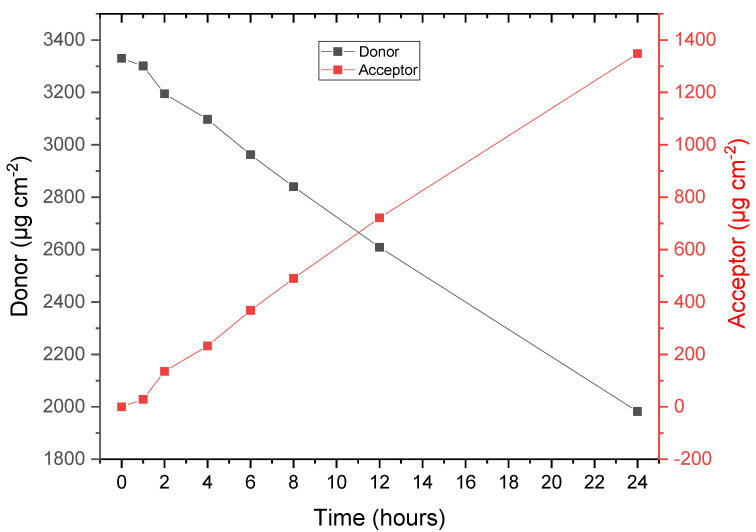
API release from formulation THQ:HPβCD.010 through a Nylon membrane.

To confirm that THQ:HPβCD is released from the BC and the release rate is not limited by the BC matrix, a passive release study was performed. This experiment was performed on a Nylon non-rate limiting membrane and PBS:PEG400 buffer was used as the acceptor solution, and it emerged, the THQ:HPβCD readily released from the BC matrix. The data presented in Figure 6 is the median mass of drug released per cm^2^ of six measurements (*n* = 6) which empirically shows that there is a stable release of API over 24 h. The extraction data is in accordance with the dose of THQ:HPβCD which was originally loaded, as shown in Table 1.

### 3.5. Adhesion

**Figure 7:**
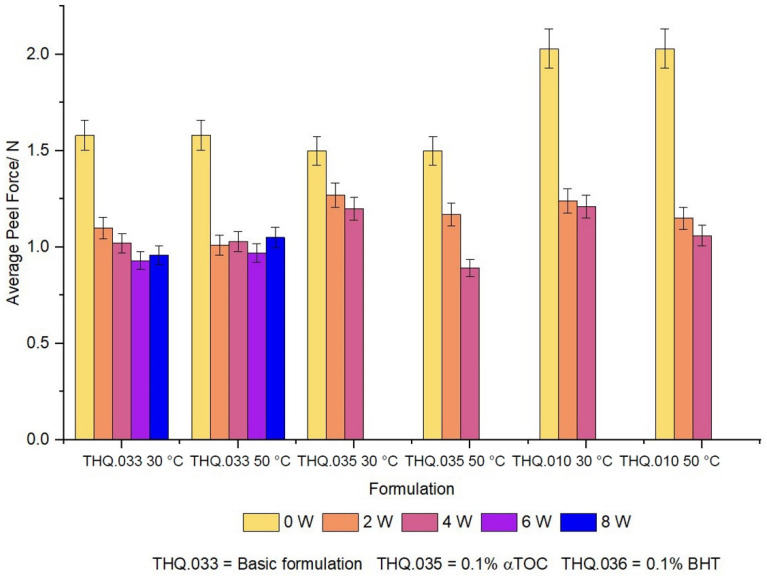
Peel adhesion of THQ:HPβCD:BC dressings at either 30 °C or 50 °C (*n* = 6, error bar = SD).

The drug delivery performance of a transdermal dressing is determined by the area in contact with the skin, as such, peel adhesion tests were performed to determine the adhesive properties of the dressings. To keep the contact area high, it is important to have good adhesive properties as any detached areas will not deliver API across the skin. The results of the peel adhesion test are outlined in Table 5 and Figure 7.

**Figure 8:**
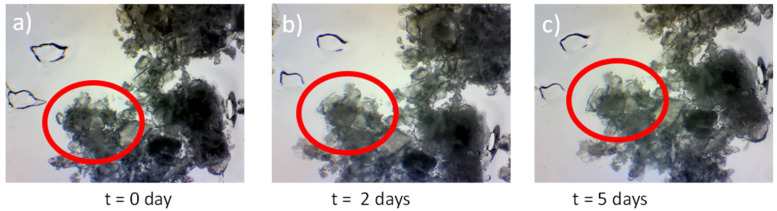
Crystal seeding images of THQ.010 at T = 0 to T = 7 days (**a**–**c**) showing that there is no crystal growth from the seed (circled) indicating that the formulation is stable.

As can be seen from Figure 8, THQ.010 has the highest average peel force of 2.1 Nbut only remains adhesive for 4 weeks at both 30 °C and 50 °C. This is in contrast with THQ.035 which has a reduced average peel force of 1.7 N, but maintains adhesive properties for the whole 8 week study. These results may offer insights into additions of excipients into the wound dressing, such as α-tocapherol, and the concessions to the mechanical properties in light of enhanced physicochemical/biological activity of the medical device.

### 3.6. Crystal Growth

To evaluate the stability of THQ:HPβCD dissolved, a crystal seeding experiment was performed. The importance of analysing the stability of the loaded dressing is to ensure that the THQ:HPβCD complex does not crystalise out of the BC- thus rendering the complex ineffective. The present study has demonstrated that the stability of the loaded dressing adheres to International Conference on Harmonisation (ICH) Q1A (R2) standards for the stability of pharmaceutically activated medical device as discussed in the European Medicines Agency guideline on quality of transdermal dressings which demands the testing of all APIs. The present study showed that no nucleation of the seeded crystal occurred over the 7-day time span [38,39].

It was observed that that formulation THQ.010 (7.0 wt% thymoquinone and 10 wt% DMSO) were found to sufficiently stable. No crystals were observed after 7 days indicating that the formulation would not recrystallize, rendering the API inactive (Figure 8). These finding are in accordance with Al-Qubaisi et al., 2019, where the authors evaluated the change in crystallinity of THQ after inclusion in HPβCD by XRD analysis [33].

## 4. Further Work

The preliminary in vitro test results advocate the potential wound management application of these hydrogels as advanced dressing materials. Further work on ex vivo and in vivo models would reveal their behavior under biological conditions. Further research aims to optimise the performance of BC hydrogels loaded with THQ to provide a responsive and controlled release delivery platform. Furthermore, in silico studies couple with gas chromatography-mass spectroscopy (GC-MS) could also be employed to determine any additional toxicity or pharmacokinetic properties of THQ against a much larger pool of cell lines. Due to contradictory information, the exact mechanism of action of THQ in wound healing is still yet not fully understood and would benefit from additional experimentation. As THQ is highly photosensitive, limitations with its application in biomedical devices became apparent. In the current study, THQ was encapsulated in HPβCD which may have improved its photodegradative behavior. However, further work needs to be done to understand its stability in the inclusion complex.

## 5. Conclusions

Phytochemicals such as thymoquinone which have been complexed with cyclodextrins have potential applications in diverse pharmaceutical fields to achieve enhanced bioavailability, solubility, stability, in vitro release and adhesive effects. The data from the present study shows that the complexation of thymoquinone and hydroxypropyl-β-cyclodextrins greatly enhanced the solubility, thus making it possible to produce an aqueous solution which can be incorporated into a bacterial cellulose matrix. It was also shown that the THQ:HPβCD:BC complex exhibits a maintained flux of the compound with an acceptable level of permeation, with an industry appropriate level of adhesion. The important findings from this feasibility study warrants further investigation with regard to advancement of formulations for potential clinical application in wound care with follow up in vivo studies. To our knowledge, no other drug delivery system to date has used bacterial cellulose in combination with cyclodextrins to both solubilise and deliver a highly hydrophobic compound, THQ, to a wound site. There have been several attempts at producing nano-emulsions and liposomal formulations as a method of drug delivery, but all have had varying results [49,50,51,52]. The main advantage our method has over nano-emulsions and liposomal formulations centers on the fact that this application aims at treating dermal wound infections. A liposomal formulation is conventionally given as a epicutaneous therapy to target systematic infection, rather than localised wounds, and a nano-emulsion historically suffers from the detrimental effects of sedimentation and poor loading uniformity [50,51,52].

## Figures and Tables

**Table 1:** Bill of materials for production of THQ:HPβCD-BC wound dressings.

Item No	Ingredient		Mass Fraction, %	Scale, mg
1	Adhesive	Dry BC	83.0	36.10
2	IC (aq)	THQ:HPβCD	7.0	3.04
3	Excipient	DMSO	10.0	4.34

**Table 2:** Solubility of THQ:HPβCD in BC with co-solvent DMSO at a mixing temperature of 80 °C.

Formulation	Dry BC, wt%	THQ:HPβCD, wt%	DMSO, wt%	Loading, Min
THQ.009	83	7	10	55
THQ.010	83	7	10	45
THQ.011	83	7	10	50
THQ.012	83	7	10	60
THQ.013	83	7	10	50

**Table 3:** Thymoquinone Release and Permeation values *.

Parameter/API	THQ:HPβCD
Release lag time, h	0
Total released amount (0–24 h), µg cm^−2^	1347.9
Average flux after lag, µg h^−1^ cm^−2^	56.2
Permeation lag time, h	2
Total permeated amount (0–24 h), µg cm^−2^	538.8
Average flux after lag, µg h^−1^ cm^−2^	22.4

* Results are median values of 6 replicates.

**Table 4:** Extraction results from thymoquinone dose determination (formulation THQ.010) *n* = 6.

Area, mAu	Extracted THQ, mg	THQ Dose, mg cm^−2^	Calculation	Dose, mg cm^−2^	±, mg cm^−2^	Variation, %
1956.65	1.67	3.32	Mean	3.31	±0.06	1.79
1982.42	1.69	3.37				
1963.84	1.67	3.33				
1969.25	1.68	3.34	Median	3.33	±0.03	0.80
1891.00	1.61	3.21				
1918.86	1.64	3.26				

**Table 5:** Peel adhesion test (90°) on thymoquinone loaded BC dressings THQ.010.

Formulation	Replicates	Max peak	Area, N/mm	Average, N/mm
THQ.010	1	3.0	220.9	2.0
	2	3.1	217.4	1.9
	3	3.4	250.3	2.2
	Average	3.2	229.6	2.1
	Std Dev	0.2	18.1	0.2
	Relative Std Dev, %	6.2	7.9,	7.9

## Data Availability

The data presented in this study are openly available.

## Supplementary Materials

The following are available online at https://www.mdpi.com/article/10.3390/pharmaceutics14122816/s1, Table S1: Percentage of liquid phase injected at each respective time point.
