# Supplementary material for: Thymoquinone: Hydroxypropyl-β-cyclodextrin Loaded Bacterial Cellulose for the Management of Wounds
Source: Pharmaceutics. 2022 Dec 15;14(12):2816. doi: 10.3390/pharmaceutics14122816 (PMC9781873; doi:10.3390/pharmaceutics14122816)
Supplement: Supplementary file 1 [file pharmaceutics-14-02816-s001.zip › pharmaceutics-2003753-S1.pdf]

pharmaceutics

MDPI

Supplementary Materials: Thymoquinone: Hydroxypropyl-β-Cyclodextrin Loaded Bacterial Cellulose for the Management of Wounds

Sam Swingler, Abhishek Gupta, Hazel Gibson, Marek Kowalczuk, Grazyna Adamus, Wayne Heaselgrave and Iza Radecka

S1. HPLC Validation

Agilent 1220 Infinity LC equipped with Variable Wavelength Detector was set at 230 nm. Data was collected and analysed using Agilent Technologies OpenLAB CDS software (version C.01.07). All chromatographic experiments were performed in the gradient mode. Flow rate was 1.5 mL min^{-1} , injection volume as 10 µL, column temperature was 40 °C. Column: Agilent Poroshell 120 EC-C18 4 µm C18 (l = 100 mm; ø = 4.6 mm) with an inline filter. Mobile phases: phase A: Water + 0.1% TFA and phase B: Methanol (table S1).

Table S1. Percentage of liquid phase injected at each respective time point.

Time (min)	Phase A (%)	Phase B (%)
0.00	50	50
2.00	50	50
5.00	30	70
5.05	50	50
7.00	50	50

Mobile phase A was prepared by adding 2.5 mL of trifluoroacetic acid to 2.5 L HPLC water. Mobile phase B was pure HPLC Methanol. Solutions were vigorously stirred for 30 min prior to application. To produce reference samples, thymoquinone (40 mg) was precisely weighed using an analytical balance into a volumetric flask before being dissolved in 20 mL of methanol/water, 50:50 vol.% (diluent). An aliquot (5.0 mL) of the obtained solution was diluted to 100 mL in the above-mentioned mixture, yielding a final stock solution (100 µg mL^{-1}). Reference samples for the validation of thymoquinone and linearity were prepared within a concentration range of 1–100 µg mL^{-1}.
